# ACL rehabilitation after reconstruction: Evidence‐based consensus recommendations from the German Knee Society—Part IV

**DOI:** 10.1002/jeo2.70875

**Published:** 2026-08-03

**Authors:** Martin Häner, Wolf Petersen, Arthur Praetorius, Natalie Mengis, Thomas Stoffels, Daniel Guenther, Thomas Pfeiffer, Julian Mehl, Andree Ellermann, Christian Eberle, Thomas Stein, Christian Schoepp, Jürgen Höher, Sven Scheffler, Amelie Stöhr, Mirko Herbort, Tobias Jung, Christoph Kittl, Philipp‐Johannes Braun, Katrin Karpinski, Andrea Achtnich, Raymond Best, Ben Wagener, Jochen Bellerich, Lena Eggeling, Tobias Gensior, Christina Valle, Janina Tennler, Sebastian Stemmler, Matthias Jäger, Lars‐Christopher Färber, Clemens Baier, Alexandra Schöllkopf, Max Wießmeier, Daniel Niederer

**Affiliations:** ^1^ Department of Orthopedics Sportsclinic Berlin, Martin Luther Hospital Berlin Germany; ^2^ Klinik für Arthroskopische Chirurgie, Sporttraumatologie und Sportmedizin, Athletikum Rhein‐Ruhr, BG Klinikum Duisburg Duisburg Deutschland; ^3^ Kantonsspital Baselland (Bruderholz) Liestal Switzerland; ^4^ OC Stadtmitte Berlin Germany; ^5^ Department of Orthopaedic Surgery, Trauma Surgery, and Sports Medicine, Cologne‐Merheim Medical Center (CMMC) Witten‐Herdecke University Cologne Germany; ^6^ Department for Orthopedic Sports Medicine Technical University Munich (TUM) Munich Germany; ^7^ Arcus Sportklinik Pforzheim Germany; ^8^ Department of Sports Medicine Goethe University Frankfurt Frankfurt am Main Germany; ^9^ SPORTHOLOGICUM® Frankfurt am Main Center for Sport and Joint Injuries Frankfurt am Main Germany; ^10^ Department of Arthroscopic Surgery, Sports Traumatology, and Sports Medicine BG Klinikum Duisburg gGmbH Duisburg Germany; ^11^ Sportsclinic Cologne Köln Germany; ^12^ Sporthopaedicum Berlin Berlin Germany; ^13^ OCM Clinic Munich Munich Germany; ^14^ Center for Musculoskeletal Surgery Charité‐University Medicine Berlin Germany; ^15^ Department of Trauma, Hand and Reconstructive Surgery Westphalian Wilhelms University Muenster Muenster Germany; ^16^ Department of Trauma and Orthopaedic Surgery BG Hospital Unfallkrankenhaus Berlin Germany; ^17^ SPORTHEUM, Sportsclinic Fellbach Germany; ^18^ Department of Sports Medicine University of Tuebingen Tuebingen Germany; ^19^ Medical Park Chiemsee Rehabilitationsklinik für Orthopädie Traumatologie und Sportmedizin Chiemsee Germany; ^20^ Department of Trauma and Orthopaedic Surgery Sports Traumatology, BG Klinikum Hamburg Hamburg Germany; ^21^ OPND, Orthopädische Praxisklinik Neuss Düsseldorf Neuss Germany; ^22^ Klinikum Poliklinik für Orthopädie und Sportorthopädie, TUM Klinikum Rechts der Isar München Germany; ^23^ Fachklinik für Orthopädie, Medical Park Chiemsee Bernau‐Felden Germany; ^24^ Medical Park Chiemsee, Rehabilitationsklinik für die Fachbereiche Orthopädie Traumatologie und Sportmedizin Bernau am Chiemsee Deutschland; ^25^ S | Medicalcenter Neckarsulm Germany; ^26^ Orthosportiv München Germany; ^27^ Orthopädie Regensburg MVZ Regensburg Germany; ^28^ Simssee‐Klinik Bad Endorf Germany; ^29^ Physiotherapie & Training Max Wießmeier Frankfurt am Main Deutschland; ^30^ Institute of Occupational, Social and Environmental Medicine Goethe University Frankfurt Germany

**Keywords:** ACL reconstruction, consensus guidelines, neuromuscular training, proprioception, rehabilitation

## Abstract

**Purpose:**

The aim of this consensus was to provide evidence‐based recommendations for individual rehabilitation interventions following anterior cruciate ligament reconstruction. These recommendations are intended to complement, rather than replace, comprehensive criterion‐based rehabilitation protocols.

**Methods:**

A modified Delphi process conducted by the German Knee Society evaluated 29 rehabilitation topics. Evidence from systematic reviews, randomized controlled trials and prospective cohort studies (A1‐C) was summarized and rated by an expert panel. Consensus was defined as ≥80% agreement between raters. Statements were graded using the Grading of Recommendations Assessment, Development and Evaluation framework, specifying the certainty of evidence.

**Results:**

Consensus was achieved for 25 topics (86%). Moderate‐certainty evidence (B2) indicates that early weight bearing likely results in safe functional recovery. Supervised and unsupervised exercise (A2–B2) likely results in improved strength and function. Rehabilitation lasting ≥9 months (A1) likely results in optimal recovery guided by functional progress. Neuromuscular training (A1) and proprioceptive training (B2) likely improve sensorimotor control and functional outcomes. Plyometric (B2) and eccentric exercises (B2), as well as blood flow restriction (A1), likely result in enhanced quadriceps strength and muscle mass. Core stability exercises (C) and aquatic therapy (B1) likely improve knee function and facilitate early return to activity. Adjunctive modalities, such as cryotherapy (A2), likely reduce pain and swelling; KT (C) and digital applications (B1) may improve early‐phase outcomes. Interventions such as whole‐body vibration training (B2) and cross‐education (C) showed inconsistent or insufficient benefits. Rehabilitation should be individualized according to patient‐specific factors, concomitant injuries and functional progress.

**Conclusion:**

These consensus recommendations provide a multimodal, evidence‐based framework for anterior cruciate ligament rehabilitation. Following these recommendations likely reduces variability in clinical practice, supports safe return to sport and might lower the risk of re‐injury.

**Level of Evidence:**

Level V, expert consensus.

## Graphical abstract

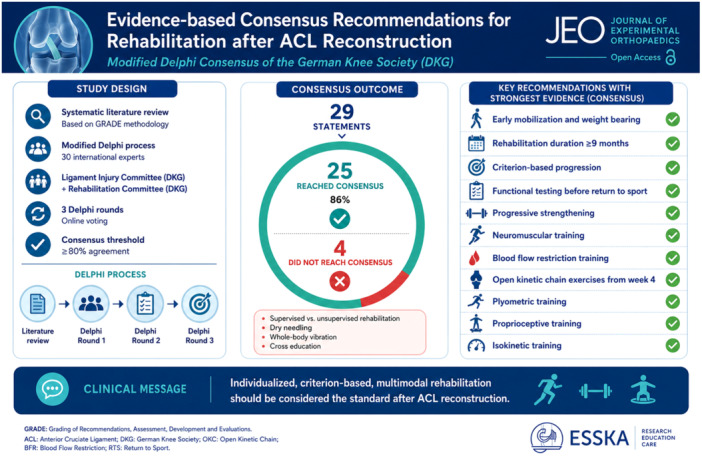

**Abbreviations**
ACL: anterior cruciate ligament
BFR: blood flow restriction training
CE: cross‐education training
CPM: continuous passive motion
DKG: German Knee Society (Deutsche Knie Gesellschaft)
DMD: digital medical device
EMG FB: Electromyographic Biofeedback
ESWT: extracorporeal shockwave therapy
GRADE: Grading of Recommendations Assessment, Development and Evaluation
KOOS: Knee Injury Osteoarthritis Outcome Score
KT: Kinesiotaping
NMES: neuromuscular electrical stimulation
NPRS: numeric pain rating scale
OKC and CKC exercises: open and closed kinetic chain exercises
PROMs: patient‐reported outcome measures
PT: proprioceptive training
TrP‐DN: trigger point dry needling

## INTRODUCTION

Anterior cruciate ligament (ACL) reconstruction is a commonly performed surgical procedure intended to restore knee stability and function following ACL injury. Postoperative rehabilitation is a crucial component of successful treatment and typically consists of a prolonged, multi‐phase process with varying therapeutic goals, interventions and progression criteria. Rehabilitation strategies may include strength training, neuromuscular and proprioceptive exercises, plyometric training and adjunctive therapeutic modalities aimed at restoring joint function and enabling a safe return to sport. Contemporary rehabilitation concepts increasingly emphasize criterion‐based progression, restoration of neuromuscular control and optimized return‐to‐sport strategies to reduce the risk of reinjury following ACL reconstruction [24]. Numerous studies have investigated different rehabilitation approaches and their impact on functional outcomes following ACL reconstruction [14, 23].

Despite the growing body of literature on ACL rehabilitation, considerable variability persists in postoperative rehabilitation protocols. Differences exist in the timing of weight‐bearing, progression of exercises, the use of supervised versus home‐based rehabilitation and the integration of adjunctive therapies [2, 45, 49]. Recent studies have further demonstrated that variations in early postoperative weight‐bearing strategies may influence biomechanical outcomes, highlighting the need for evidence‐based recommendations regarding rehabilitation progression after ACL reconstruction [59]. These variations may lead to inconsistent clinical practice and may negatively influence patient outcomes, including return‐to‐sport rates and risk of reinjury [27].

To address this lack of consistency, the development of evidence‐based and consensus‐driven clinical practice guidelines is essential. Previous guideline initiatives and systematic reviews have highlighted the importance of integrating current scientific evidence into structured rehabilitation pathways to support clinicians and therapists in clinical decision‐making [2, 45].

By establishing a standardized and evidence‐informed approach, rehabilitation strategies may help improve patient outcomes, reduce the risk of reinjury and facilitate a safe return to physical activity following ACL reconstruction. Consequently, the aim of the present consensus was to develop evidence‐based clinical recommendations for rehabilitation after ACL reconstruction. These recommendations are intended to complement, rather than replace, comprehensive criterion‐based rehabilitation protocols.

## MATERIALS AND METHODS

**Figure 1:**
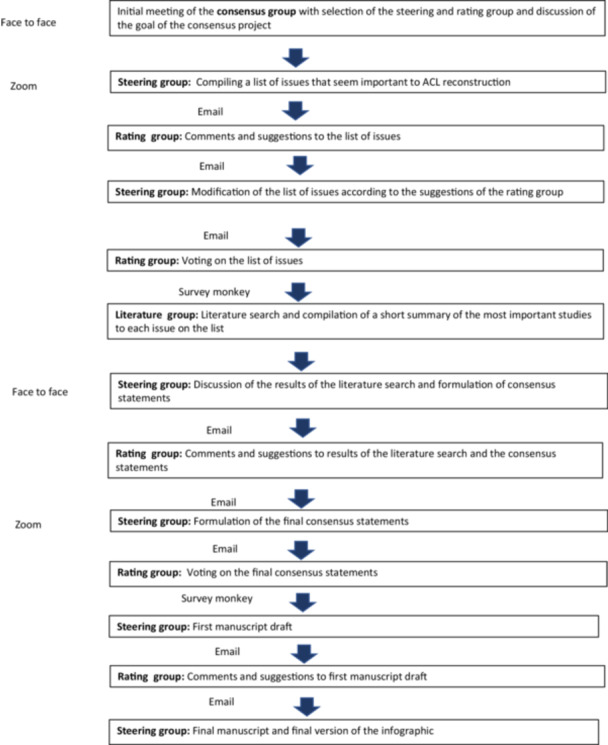
Schematic drawing of the procedure during the consensus process.

To develop evidence‐based rehabilitation recommendations following ACL reconstruction, a modified Delphi process was conducted (Figure 1). This project was initiated by the Ligament Injuries and Rehabilitation Committee of the German Knee Society (Deutsche Knie Gesellschaft, DKG) and followed current recommendations for developing and reporting clinical consensus statements [7].

The methodology was developed in accordance with current recommendations for Delphi‐based consensus studies. Additional details regarding panel composition, literature review, statement development and voting procedures have been added to improve transparency.

An expert panel consisting of 30 experts was assembled from the German Knee Society, including orthopaedic knee surgeons, sports medicine physicians and rehabilitation specialists with extensive clinical and academic experience in ACL reconstruction and rehabilitation.

The recommendations primarily address rehabilitation following ACL reconstruction. Concomitant injuries were considered where sufficient evidence was available and addressed in specific statements.

A modified Delphi methodology, based on the classical Delphi design, was applied over three predefined rounds [17, 28]. The modifications consisted of structured online surveys combined with an in‐person discussion before the final Delphi round to clarify ambiguities and facilitate iterative refinement.

The project was coordinated by a steering group of two senior orthopaedic knee surgeons (M. H. and W. P.). The initial questionnaire was drafted, scientific statements were prepared, and each Delphi round was supervised by the steering group.

The steering group was responsible for coordinating the consensus process, preparing the questionnaires and evidence summaries and had no influence on the anonymous voting.

The initial open‐ended survey addressed key aspects of ACL rehabilitation (e.g., early weight bearing, supervised/unsupervised exercise, rehabilitation duration, blood flow restriction). The literature search and evidence summaries were prepared by the steering group before presentation to the expert panel.

A systematic literature review (MEDLINE, Scopus, CENTRAL; 1996–2026) identified relevant human studies, with animal and cadaveric data used for contextual information. Inclusion and exclusion criteria and screening procedures followed standard systematic review methodology.

Based on survey responses and the literature, 29 statements were formulated and categorized into three domains: general rehabilitation, assistive devices and exercise‐based therapy.

For each statement, the available body of evidence was first categorized according to study design. Subsequently, the certainty of the evidence was assessed using the Grading of Recommendations Assessment, Development and Evaluation (GRADE) framework, taking into account risk of bias, inconsistency, indirectness, imprecision and publication bias [6, 17, 28, 62]. Where appropriate, upgrading factors such as large effect sizes were also considered.

To facilitate interpretation within the Delphi process, GRADE levels were translated into an A–E grading system (Table 1): Grade A corresponded to high certainty of evidence, Grade B to moderate certainty, Grade C to low certainty and Grade D to very low certainty. Statements based primarily on expert opinion due to limited or inconsistent evidence were classified as Grade E.

**Table 1:** Evidence categories used for grading the available literature.

Grade	Type of study
A1	Several (>2) Level 1, randomized clinical studies with similar results or a meta‐analysis
A2	Single Level 1 randomized clinical trial or systematic review of Level 1 studies
B1	Prospective cohort study, non‐randomized controlled trial with comparison group
B2	Single randomized clinical trial, prospective cohort study or any comparison group that is not Level 1
C	Case series
D	Case report
E	Expert opinion/basic research

*Note*: The grading system reflects the methodological quality of the highest level of evidence supporting each recommendation, ranging from A1 (highest) to E (expert opinion/basic research).

All statements were rated by the panel on a 5‐point Likert scale (1 = strongly disagree to 5 = strongly agree). Ratings of 4 and 5 were classified as agreement, whereas ratings of 1–3 were classified as disagreement. Consensus was predefined as ≥80% agreement.

Final approval of consensus statements was obtained via a secure online voting platform (https://www.surveymonkey.de), with anonymized data processing.

### Statistical analysis

The data were processed using SPSS (Version 20.0; IBM) and Excel 2019 (Microsoft Corporation).

## RESULTS

Twenty‐nine statements were evaluated over three Delphi rounds. Consensus (≥80%) was achieved for 25 statements, whereas four statements did not reach the predefined threshold. Topics were categorised into three predefined subgroups: (A) general aspects of rehabilitation, (B) assistive devices and (C) exercise‐based training therapy. All recommendations are summarized in Table 2.

**Table 2:** Evidence summary—ACL reconstruction rehabilitation.

#	Recommendation	Level of evidence	Certainty	Likely effect/Outcome
1	Partial weight bearing for 2 weeks after isolated ACL reconstruction	B2	Moderate	Likely results in safe early loading without increased revision
2	Extended partial weight bearing in presence of concomitant injuries	C	Low	May result in improved outcomes
3	Unsupervised rehabilitation	B2	Moderate	Might result in equivalent outcomes compared to supervised rehabilitation
4	Combination of supervised and unsupervised rehabilitation	A2	Moderate	Likely results in improved outcomes
5	Rehabilitation duration of at least 9 months	A1	High	Likely results in optimal recovery based on patient progress and intensity
6	Completion of rehabilitation based on functional criteria	A1	Moderate	Likely results in better individualized recovery than time‐based alone
7	Postoperative psychological and social interventions	E	Very low	Might result in improved outcomes in some patients
8	Hard‐frame knee braces after isolated ACL‐Rx	B1	Moderate	Likely not required
9	Hard‐frame knee braces with concomitant meniscus injury	C	Low	May result in improved outcomes
10	Kinesiotaping (KT) in early phase	B2	Low	May reduce pain and swelling, effects inconsistent
11	Cryotherapy during first 3 days	A2	Moderate	Likely results in reduced pain and swelling; compressive cryotherapy may be more effective
12	Continuous passive motion	A2	Moderate	Might not result in meaningful improvement
13	Extracorporeal shockwave therapy	B2	Low	Cannot be recommended for routine rehabilitation
14	Digital applications for rehabilitation	B1	Moderate	Likely results in improved KOOS scores and pain outcomes
15	Neuromuscular electrical stimulation (NMES)	A1	Moderate	Likely results in improved quadriceps strength and functional outcomes
16	EMG biofeedback	A1	Moderate	Likely results in improved muscle activation and outcomes
17	Blood flow restriction with low‐load resistance training	A1	Moderate	Likely results in improved strength and muscle mass
18	Whole‐body vibration training	B2	Low	Cannot be generally recommended; benefits inconsistent
19	Dry needling in early rehab phase	C	Very low	Might result in improved early quadriceps function as adjunct therapy
20	Early open kinetic chain exercises from Week 4	A2	Moderate	Likely results in improved quadriceps strength
21	Eccentric exercises	B2	Moderate	Likely results in improved strength recovery
22	Isokinetic training	B2	Moderate	Likely results in strength recovery, especially early/mid rehabilitation phases
23	High‐intensity training	B2	Moderate	Likely results in improved muscle strength without compromising joint stability
24	Proprioceptive training	B2	Moderate	Likely results in improved strength and sensorimotor function
25	Plyometric training	B2	Moderate	Likely results in improved functional outcomes
26	Cross‐education training	C	Low	Cannot currently be recommended due to conflicting results
27	Core stability exercises	C	Low	Likely results in improved core control and support for rehabilitation
28	Aquatic therapy	B1/C	Moderate	Likely results in improved ROM, knee function, early return to activity
29	Neurocognitive training	C	Low	May result in improved load symmetry and step pattern normalization

*Note*: This document summarizes the 29 key recommendations for rehabilitation after ACL reconstruction, including Level of Evidence (A1–E), Certainty (High/Moderate/Low/Very Low) and the Likely Effect/Outcome according to GRADE standards.

Abbreviations: ACL, anterior cruciate ligament; KOOS, Knee injury and Osteoarthritis Outcome Score.

### A. General aspects of rehabilitation

#### Postoperative weight bearing

Questions:

1. Is early full weight bearing after ACL reconstruction supported by evidence, and should it be recommended?
2. Should partial weight bearing be prolonged in the presence of concomitant injuries?

Literature overview:

Current clinical practice guidelines and clinical studies suggest that early full weight bearing after ACL reconstruction is feasible and may facilitate faster recovery of knee function and muscle strength, enabling an earlier return to sport [2, 74, 75]. Several studies reported no significant differences in patient‐reported outcome measures (PROMs) between early and delayed weight‐bearing protocols [15, 39].

However, some evidence indicates potential disadvantages of accelerated rehabilitation, including trends toward higher revision rates or increased tunnel widening, although these differences were not consistently clinically relevant [15, 37, 77]. In contrast, selected studies reported superior mid‐term outcomes with short‐term partial or non–weight‐bearing protocols [36].

Overall, weight‐bearing recommendations after ACL reconstruction should be individualised, particularly in the presence of concomitant injuries.

Statement 1:

Based on moderate‐certainty evidence, partial weight bearing for 2 weeks is reasonable due to a trend toward increased tunnel widening, while revision rates are not significantly elevated.

GRADE Evidence category: B2

Certainty of the evidence: Moderate

Agreement: 90%

Statement 2:

Based on low‐certainty evidence, extended partial weight bearing (>2 weeks) may be considered in the presence of concomitant injuries, such as cartilage, collateral ligament or meniscus lesions.

GRADE Evidence category: C

Certainty of the evidence: Low

Agreement: 100%

#### Unsupervised versus supervised rehabilitation

Questions:

1. Is unsupervised rehabilitation equivalent to supervised rehabilitation after ACL reconstruction?
2. Should supervised rehabilitation be complemented by unsupervised exercises?

Literature overview:

Unsupervised rehabilitation is defined as a structured, periodised programme performed at home or in other settings (e.g., fitness centre) without direct physiotherapist supervision. Multiple studies showed no differences between unsupervised and supervised programmes regarding knee laxity, subjective function, functional outcomes, muscle strength or atrophy [19, 25, 26, 32, 41, 63, 64, 74].

Statement 3:

Based on moderate‐certainty evidence, unsupervised rehabilitation might be equivalent to supervised rehabilitation.

GRADE Evidence category: B2

Certainty of the evidence: Moderate

Agreement 36%

Statement 4:

Based on moderate‐certainty evidence, a combination of supervised and unsupervised rehabilitation likely improves outcomes.

GRADE Evidence category: A2

Certainty of the evidence: Moderate

Agreement: 97%

#### Rehabilitation duration

Question:

Is there an optimal duration of rehabilitation after ACL reconstruction, and can a specific timeframe be generally recommended?

Literature overview:

Van Melick et al. [45], in a systematic review, reported that rehabilitation should be functional, sport‐specific and focused on return to sport. Assessment of rehabilitation progress and readiness to return should include strength, jump, range‐of‐motion and psychological testing. Recommended rehabilitation duration is 9–12 months. Similarly, Grindem et al. [27] demonstrated that a minimum of 9 months of rehabilitation reduces the risk of re‐rupture.

Statement 5:

Based on moderate‐certainty evidence, rehabilitation programmes should last at least 9 months, depending on patient intensity and progress.

GRADE Evidence category: A1

Certainty of the evidence: Moderate

Agreement: 90%

Statement 6:

Based on moderate‐certainty evidence, completion of rehabilitation should be based on functional criteria rather than time alone.

GRADE Evidence category: A1

Certainty of the evidence: Moderate

Agreement: 97%

#### Psychological and social interventions

Question: Do psychological and social interventions improve rehabilitation outcomes after ACL reconstruction?

Literature overview:

Results on the additional benefits of psychological and social interventions for improving postoperative function, pain or self‐efficacy are inconsistent. Evidence is limited regarding improvements in postoperative quality of life, anxiety or fear of re‐injury. No study has investigated the effect of psychosocial interventions on return to sport or activity [13, 33].

Statement 7:

Based on very low‐certainty evidence, postoperative psychological and social interventions might improve outcomes, but cannot be generally recommended.

GRADE Evidence category: E

Certainty of the evidence: very low

Agreement: 73%

### B. Assistive devices

#### Knee braces

Questions:

1. Is the use of hard‐frame knee braces recommended after isolated ACL reconstruction?
2. Are there indications for using hard‐frame knee braces in ACL reconstruction with concomitant injuries?

Literature overview:

Yang et al. [76] compared clinical outcomes after ACL reconstruction in patients with and without a brace and found no significant differences in objective International Knee Documentation Committee, Lysholm, Tegner scores, side‐to‐side difference, single‐leg hop test or visual analogue scale pain scores. Similarly, a recent prospective randomized study reported no differences between rehabilitation with or without a brace [65].

Regarding concomitant injuries, most authors recommend hard‐frame braces. No clear benefit of bracing was observed for isolated meniscus injuries or combined ACL–meniscus injuries [43]. However, a recent study from France suggested that limiting flexion may be beneficial during rehabilitation of surgically treated meniscus injuries [18].

Statement 8:

Based on moderate‐certainty evidence, hard‐frame knee braces are generally not required after isolated ACL reconstruction

GRADE Evidence category: B1

Certainty of the evidence: Moderate

Agreement: 97%

Statement 9:

Based on low‐certainty evidence, hard‐frame braces may be considered after ACL reconstruction with concomitant meniscus injury.

GRADE Evidence category: C

Certainty of the evidence: Low

Agreement: 97%

#### Kinesiotaping (KT)

Question:

Can KT be used after ACL reconstruction, and in which phase is it appropriate?

Literature overview:

A meta‐analysis demonstrated that adding KT to standard postoperative physiotherapy reduces pain and knee swelling during the early phase after ACL reconstruction [4].

Statement 10:

Based on low‐certainty evidence, KT may reduce pain and swelling in the early phase after ACL reconstruction, but effects are inconsistent.

GRADE Evidence category: B2

Certainty of the evidence: Low

Agreement: 58%

#### Cryotherapy/Compressive cryotherapy

Question:

Can cryotherapy be used after ACL reconstruction, and are there any differences in application methods?

Literature overview:

Systematic reviews indicate that cryotherapy reduces medication use, subjective pain and improves patient satisfaction during the first 3 days postoperatively [14, 23, 68] optimal method of application remains unclear. A meta‐analysis showed that compressive cryotherapy further reduces medication use and pain compared with cryotherapy alone [68].

Statement 11:

Based on moderate‐certainty evidence, cryotherapy is recommended during the first 3 days after ACL reconstruction; compressive cryotherapy may be more effective.

GRADE Evidence category: A2

Certainty of the evidence: Moderate

Agreement: 97%

#### Continuous passive motion (CPM)

Question:

Can CPM be used after ACL reconstruction, and is it recommended?

Literature overview:

A 2019 meta‐analysis showed that CPM has positive effects on pain relief during the first 2 postoperative days, on knee flexion in Weeks 1–6, and on swelling between Weeks 4–6 postoperatively [34]. In contrast, Wright et al. reported no significant benefit of CPM after ACL reconstruction [74].

Statement 12:

Based on moderate‐certainty evidence, CPM cannot be routinely recommended after ACL reconstruction.

GRADE Evidence category: A2

Certainty of the evidence: Moderate

Agreement: 97%

#### Extracorporeal shockwave therapy (ESWT)

Question:

Can ESWT be used to support rehabilitation after ACL reconstruction?

Literature overview:

Shin et al. [67], in a meta‐analysis, found that ESWT combined with standard rehabilitation led to improved patient‐reported outcomes. However, these differences do not appear to be clinically relevant.

Statement 13:

Based on low‐certainty evidence, ESWT cannot be recommended for rehabilitation after ACL reconstruction.

GRADE Evidence category: B2

Certainty of the evidence: Low

Agreement: 100%

#### Digital applications

Question:

Can digital applications be used to support rehabilitation after ACL reconstruction, and should they complement supervised physiotherapy?

Literature overview:

Schmidt et al. [64] reported that a medical device app was safe and effective; combining the app with standard care significantly improved prehabilitation and postoperative rehabilitation outcomes compared with standard care alone, including Knee Injury Osteoarthritis Outcome Score (KOOS) and numeric pain rating scale (NPRS). Dunphy et al. [16] found that a digital application used alongside supervised physiotherapy was acceptable to patients, improved confidence and motivation and identified opportunities for future development. Mengis et al. [46] reported no adverse events associated with a sensor‐based digital medical device (DMD), confirming its clinical validity for objective knee function assessment. Zhou et al. [78] demonstrated that digital therapy supports postoperative rehabilitation after ACL reconstruction.

Statement 14:

Based on moderate‐certainty evidence, digital applications can effectively support pre‐ and postoperative rehabilitation after ACL reconstruction and likely improve KOOS scores and pain outcomes.

GRADE Evidence category: B1

Certainty of the evidence: Moderate

Agreement: 97%

### C. Exercise‐based training therapy

#### Neuromuscular electrical stimulation (NMES)

Question:

Can NMES be used after ACL reconstruction, and is it recommended?

Literature overview:

Recent systematic reviews have shown that NMES is effective in improving quadriceps strength when applied alone or in combination with rehabilitation exercises [3, 23].

Statement 15:

Based on moderate‐certainty evidence, NMES can be recommended after ACL reconstruction.

GRADE Evidence category: A1

Certainty of the evidence: Moderate

Agreement: 93%

#### Electromyographic biofeedback (EMG FB)

Question:

Can EMG biofeedback be used after ACL reconstruction, and is it recommended?

Literature overview:

Systematic reviews have shown that EMG biofeedback improves quadriceps strength and knee extension after ACL reconstruction [1].

Statement 16:

Based on moderate‐certainty evidence, EMG biofeedback can be recommended after ACL reconstruction.

GRADE Evidence category: A1

Certainty of the evidence: Moderate

Agreement: 93%

#### Blood flow restriction training (BFR)

Question:

Can BFR be used after ACL reconstruction, and is it recommended?

Literature overview:

BFR involves applying a specialized cuff around the thigh to restrict blood flow through automatic or manual compression. BFR therapy is intended to stimulate muscle anabolism, allowing low‐load exercises to achieve effects comparable to high‐load training. Recent systematic reviews have shown that, compared with standard rehabilitation alone, BFR after ACL reconstruction has positive effects on muscle strength, muscle size, pain and PROMs [8, 11, 22, 42].

Statement 17:

Based on moderate‐certainty evidence, BFR combined with low‐load resistance training can be recommended after ACL reconstruction.

GRADE Evidence category: A1

Certainty of the evidence: Moderate

Agreement: 100%

#### Whole‐body vibration training

Question:

Can whole‐body vibration training be used after ACL reconstruction, and is it recommended?

Literature overview:

A recent meta‐analysis showed that adding whole‐body vibration therapy to standard postoperative rehabilitation after ACL reconstruction does not significantly improve lower‐extremity strength or anterior‐posterior stability, but may improve mediolateral and overall postural stability [60]. Another study by Kotsifaki et al. [38] found no effects on range of motion, proprioception or subjective knee function.

Statement 18:

Based on low‐certainty evidence, whole‐body vibration training cannot be generally recommended after ACL reconstruction.

GRADE Evidence category: B2

Certainty of the evidence: Low

Agreement: 93%

#### Trigger point dry needling (TrP‐DN)

Question:

Can dry needling be recommended after ACL reconstruction?

Literature review:

A recent RCT showed that quadriceps vastus medialis TrP‐DN combined with a rehabilitation protocol in patients after ACL reconstruction increased range of motion (ROM) and functionality (7–21 days postoperatively) [72].

Statement 19:

Based on very low‐certainty evidence, dry needling might improve early quadriceps function and can be considered as an adjunct therapy.

GRADE Evidence category: C

Certainty of the evidence: Very low

Agreement: 33%

#### Open and closed kinetic chain exercises (OKC and CKC exercises)

Question: Can open kinetic chain exercises be initiated from Week 4 after ACL reconstruction?

Literature review:

A meta‐analysis showed that OKC exercises are superior to CKC exercises in improving quadriceps strength and reducing knee laxity [52].

Two RCTs demonstrated that OKC exercises between 45° and 90° of flexion can be safely implemented as early as the fourth postoperative week [31, 66].

A recently published systematic review also confirmed the effectiveness of OKC exercises [20].

Statement 20:

Based on moderate‐certainty evidence, early OKC exercises from Week 4 likely improve quadriceps strength after ACL reconstruction.

GRADE Evidence category: A2

Certainty of the evidence: Moderate

Agreement: 100%

#### Eccentric training

Question: Should eccentric exercises be used in rehabilitation after ACL reconstruction?

Literature overview:

Friedmann‐Bette et al. [21]: Combined concentric/eccentric training with eccentric overload leads to significantly greater muscle hypertrophy compared with conventional concentric/eccentric training.

Stojanović et al. [70]: Eccentric strength training in the late phase of ACL rehabilitation, performed two to three times per week over 6 weeks, results in better outcomes in professional team‐sport athletes regarding leg strength, vertical jump performance and single‐ and triple‐hop tests of the injured limb compared with conventional strength training.

Milandri et al. [47]: In ACL rehabilitation, progressive eccentric cycling training in male patients was not clinically more effective than concentric training.

Kasmi et al. [35]: Combined eccentric/plyometric training appears to be the most effective training method, as it positively affects stability and functional performance during rehabilitation after ACL surgery.

Statement 21:

Based on moderate‐certainty evidence, eccentric exercises may be incorporated as part of rehabilitation after ACL reconstruction.

GRADE Evidence category: B2

Certainty of the evidence: Moderate

Agreement: 97%

#### Isokinetic training

Question:

Should isokinetic training be used in rehabilitation after ACL reconstruction?

Literature overview:

Vidmar et al. [73]: Isokinetic eccentric training produces greater improvements than conventional eccentric training in quadriceps muscle mass and strength in recreational athletes after ACL reconstruction.

Petrucci et al. [56]: Isokinetic training can be integrated into rehabilitation after ACL reconstruction and may support restoration of hamstring–quadriceps strength balance, particularly in the early and mid phases.

Statement 22:

Based on moderate‐certainty evidence, isokinetic training can be recommended for strength recovery, especially in the early and mid rehabilitation phases after ACL reconstruction.

GRADE Evidence category: B2

Certainty of the evidence: Moderate

Agreement: 100%

#### Low‐ versus high‐intensity resistance training

Question:

Can high‐intensity training be used during rehabilitation after ACL reconstruction?

Literature overview:

Bieler et al. [5] reported that high‐intensity resistance training during rehabilitation after ACL reconstruction can improve muscle strength without negatively affecting joint laxity.

Statement 23:

Based on moderate‐certainty evidence, high‐intensity training can be used during rehabilitation and likely improves muscle strength without compromising joint stability.

GRADE Evidence category: B2

Certainty of the evidence: Moderate

Agreement: 97%

#### Proprioceptive training (PT)

Question:

Can PT be used during rehabilitation after ACL reconstruction?

Literature overview:

Liu‐Ambrose et al. [44] showed that proprioceptive training alone can lead to increases in isokinetic strength, and restoration of quadriceps strength is important for maximizing knee function after surgery.

Cooper et al. [12] found benefits in strength and proprioception outcomes in the proprioceptive training group, although no advantages were observed in activity‐based measures. No harmful effects, such as increased passive joint laxity or strength loss, were reported compared with standard rehabilitation programmes.

Statement 24:

Based on moderate‐certainty evidence, proprioceptive training should be included in rehabilitation as it likely improves strength and sensorimotor function.

GRADE Evidence category: B2

Certainty of the evidence: Moderate

Agreement: 100%

#### Plyometric training

Question:

Should plyometric exercises be included in rehabilitation after ACL reconstruction?

Literature overview:

Five studies compared neuromuscular training programmes including plyometrics, flexibility and sport‐specific exercises with conventional rehabilitation protocols (including strength training) [35, 36, 58, 60, 69, 70]. One study additionally evaluated the combination of plyometric and eccentric training [35]. Another study examined an 8‐week programme of low‐ and high‐intensity plyometric exercises (running, jumping, flexibility) on knee function, cartilage metabolism and other clinically relevant outcomes [10].

Statement 25:

Based on moderate‐certainty evidence, plyometric training can be integrated into rehabilitation after ACL reconstruction.

GRADE Evidence category: B2

Certainty of the evidence: Moderate

Agreement: 100%

#### Cross‐education training (CE)

Question:

Should CE be incorporated in rehabilitation after ACL reconstruction?

Literature overview:

Seven studies investigated contralateral strength training and its effect on the injured limb after ACL surgery. Results were inconsistent: CE showed a positive effect on knee function in the early phase, while effects on quadriceps strength in early and mid‐phase rehabilitation were mixed. No effects were observed in the late rehabilitation phase. CE had no impact on hamstring strength, single‐leg hop, balance or proprioception [30, 48, 53, 54, 55, 79, 80].

Statement 26:

Based on low‐certainty evidence, CE cannot currently be recommended due to conflicting results.

GRADE Evidence category: C

Certainty of the evidence: Low

Agreement: 91%

#### Core stability training

Should core stability exercises be included in rehabilitation after ACL reconstruction?

Literature overview:

Two studies investigated the addition of a core stability exercise programme to conventional rehabilitation protocols [50, 61]. These studies demonstrated that incorporating core stability exercises may improve subjective knee function and range of motion but did not provide additional benefits regarding pain.

Statement 27:

Based on low‐certainty evidence, core stability exercises can be integrated into rehabilitation after ACL reconstruction.

GRADE Evidence category: C

Certainty of the evidence: Low

Agreement: 100%

#### Aquatic therapy

Question:

Should aquatic therapy be incorporated into rehabilitation after ACL reconstruction?

Literature overview:

- Peultier‐Celli et al. [57]: An innovative aquatic rehabilitation programme allowed faster recovery and earlier return to social, sports and occupational activities.
- Hajouj et al. [29]: Integrating aquatic proprioceptive training into conventional accelerated rehabilitation improved proprioceptive efficiency after ACL reconstruction.
- Tovin et al. [71]: The aquatic group showed higher Lysholm scores than the land‐based group. No differences were observed in knee PROM, thigh girth or quadriceps strength. Joint effusion was lower in the aquatic group after 8 weeks, while the land group achieved higher peak torque for isokinetic knee flexion.
- Li et al. [40]: Water‐based walking exercises after ACL reconstruction led to greater improvements in quadriceps strength, proprioception and overall knee performance compared to land‐based training.

Statement 28:

Based on moderate‐certainty evidence, aquatic therapy can be used in the early phase after wound healing to likely improve subjective knee function.

GRADE Evidence category: B1

Certainty of the evidence: Low

Agreement: 93%

#### Neurocognitive training

Question: Should neurocognitive training be incorporated into rehabilitation after ACL reconstruction?

Literature overview:

One randomized controlled trial demonstrated that a neurocognitive rehabilitation approach can be an effective adjunct after ACL reconstruction. The training group showed faster load symmetry, reduced step width and more rapid resolution of oedema [9].

Statement 29:

Based on low‐certainty evidence, neurocognitive training can be integrated as an additional intervention in ACL reconstruction rehabilitation.

GRADE Evidence category: C

Certainty of the evidence: Low

Agreement: 100%

## DISCUSSION

This consensus project provides a comprehensive overview of rehabilitation following ACL reconstruction, with evidence‐graded recommendations based on the literature and expert panel agreement. All interventions should be applied within a criterion‐based rehabilitation programme according to the patient's functional recovery and rehabilitation stage.

There is moderate‐certainty evidence (B2) that early mobilisation and weight‐bearing likely result in improved functional recovery, with partial weight bearing recommended for the first 2 weeks after isolated ACL reconstruction. Low‐certainty evidence (C) suggests that extended partial weight bearing may result in improved outcomes in the presence of concomitant injuries, such as meniscus or collateral ligament lesions [36, 51, 74, 75]. Moderate‐certainty evidence (B2) indicates that accelerated rehabilitation may result in earlier functional recovery, although potential risks, such as tunnel widening, should be considered [15, 37, 77]. Rehabilitation duration should generally extend to at least 9 months, with high‐certainty evidence (A1) that progression guided by functional criteria likely results in optimal recovery, rather than following fixed timelines [27, 45].

Moderate‐certainty evidence (B1/B2) shows that hard‐frame knee braces are generally not required after isolated ACL reconstruction, but low‐certainty evidence (C) suggests that braces may result in improved outcomes when concomitant injuries are present [18, 40, 43, 65, 76]. Moderate‐certainty evidence (A2/B2) indicates that early‐phase interventions such as KT and cryotherapy likely result in reduced pain and swelling [4, 13, 14, 23, 33, 34, 67]. Conversely, moderate‐ to very low‐certainty evidence (A2/B2/E) suggests that CPM, ESWT and psychosocial interventions might not result in clinically meaningful improvements and cannot be routinely recommended [4, 13, 14, 23, 33, 34, 67]. Moderate‐certainty evidence (B1) shows that digital applications likely result in improved patient‐reported outcomes and engagement when used for rehabilitation [16, 46, 64, 78].

Exercise‐based training remains the cornerstone of ACL rehabilitation. There is moderate‐ to high‐certainty evidence (A1/B2) that NMES, EMG biofeedback, blood flow restriction (BFR) and high‐intensity resistance training likely result in improved quadriceps strength, muscle mass and functional outcomes [1, 3, 5, 8, 11, 22]. Moderate‐certainty evidence (A2/B2) indicates that open kinetic chain (OKC) exercises from Week 4, eccentric exercises and isokinetic training likely result in enhanced strength recovery and joint function [20, 21, 23, 31, 47, 52, 56, 73]. Moderate‐certainty evidence (B2) shows that proprioceptive and plyometric exercises likely result in improved sensorimotor control, balance and subjective knee function [12, 35, 44, 58, 69]. Low‐ to moderate‐certainty evidence (B2/C) suggests that whole‐body vibration training and cross‐education may not result in consistent benefits and are not broadly recommended [38, 48, 60, 79, 80]. Moderate‐certainty evidence (B1/C) indicates that core stability exercises and aquatic therapy likely result in improved range of motion, knee function and early return to activity [29, 40, 50, 57, 61]. Low‐certainty evidence (C) suggests that neurocognitive training may result in accelerated normalization of load symmetry and gait patterns [9], whereas very low‐certainty evidence (E) indicates that dry needling might result in improved early quadriceps function as an adjunct therapy, although supporting evidence remains limited [72].

Successful return to sport additionally requires sport‐specific progression, load management, movement quality and functional testing, which were beyond the scope of the present consensus.

Overall, these findings emphasize a multimodal, individualized rehabilitation approach, with a focus on progressive strength, neuromuscular, proprioceptive and functional training and selective use of adjunctive modalities. Clinical decisions should be tailored to patient‐specific factors, including concomitant injuries, preoperative fitness and psychosocial readiness.

Consensus was not achieved for four statements, likely reflecting both limited or conflicting evidence and variability in clinical practice. In particular, interventions supported by low or very low certainty of evidence resulted in greater disagreement among panel members. A potential source of bias is the composition of the expert panel, which predominantly consisted of clinicians with extensive experience in structured, supervised rehabilitation settings. This may have influenced voting behaviour, particularly for topics such as unsupervised versus supervised rehabilitation, where current evidence suggests comparable outcomes but may conflict with established clinical practice. These findings highlight the need to interpret non‐consensus statements with caution and emphasize the importance of further high‐quality research to better define the role of these interventions in ACL rehabilitation.

Limitations of this consensus include heterogeneity of study designs, outcome measures and low levels of evidence for several interventions, particularly psychosocial support and dry needling. Further high‐quality RCTs are warranted to determine optimal rehabilitation progression, evaluate combined intervention effects and assess long‐term outcomes, including reinjury rates and return‐to‐sport metrics. Another limitation is that the consensus focused on individual rehabilitation interventions rather than comprehensive programme design. Therefore, specific recommendations regarding intervention timing, dosage, progression criteria and rehabilitation staging could not be formulated.

A further limitation inherent to consensus‐based methodologies is the composition of the expert panel, as the professional background and clinical experience of the participating experts may introduce a degree of subjectivity or bias toward specific perspectives.

Furthermore, the composition of the expert panel may have introduced selection bias despite the multidisciplinary expertise of the participants.

## CONCLUSION

In conclusion, this consensus provides a structured, evidence‐informed framework for rehabilitation after ACL reconstruction. Integrating progressive, functional and multimodal training with targeted adjunctive interventions may optimize recovery, improve patient‐reported outcomes and reduce variability in clinical practice.

## AUTHOR CONTRIBUTIONS

All authors have made substantial contributions to the conception and design, acquisition of data and analysis and interpretation of data.

## FUNDING INFORMATION

The authors have no funding to report.

## CONFLICT OF INTEREST STATEMENT

Mirco Herbort is a consultant for Medacta International, Arthrex, Stryker and Enovis and receives royalties from Medacta International. Thomas Stoffels is a consultant for OPED. Wolf Petersen is a consultant for Karl Storz, Stryker, Geistlich and Arthrex. Julian Mehl is a consultant for Arthrex and Enoviscos. Daniel Guenther is a consultant for Arthrex, Stryker, Karl Storz and Medical Magnesium; receives lecture fees from Geistlich, Codon, Rimasys, Medi, Anika and Telos; and receives travel support from Smith & Nephew. Andrea Achtnich is a consultant for Arthrex. Martin Häner receives lecture fees from Sporlastic and Aspen. The remaining authors declare no conflict of interest.

## ETHICS STATEMENT

No ethical approval was required for this consensus study, as it involved only voluntary surveys of expert panel members and did not include patient data.

## Data Availability

Raw data can be made available upon request.
